# Male Occult Primary Breast Cancer Diagnosed with Small Bowel Metastases: A Case Report

**DOI:** 10.70352/scrj.cr.24-0089

**Published:** 2025-05-16

**Authors:** Suguru Ogata, Uhi Toh, Kunihiro Ozaki, Yutaro Mihara, Nanae Ogata, Yuko Takao, Shuko Saku, Rie Sugihara, Fumihiko Fujita

**Affiliations:** 1 Department of Surgery, Kurume University School of Medicine, Kurume, Fukuoka, Japan; 2 Department of Surgery, Saiseikai Hita Hospital, Hita, Oita, Japan; 3 Department of Pathology, Kurume University School of Medicine, Kurume, Fukuoka, Japan

**Keywords:** occult breast cancer, male breast cancer, lobular carcinoma, small bowel metastasis, brain metastases

## Abstract

**INTRODUCTION:**

Male occult breast cancers are extremely rare and often difficult to diagnose. With only few cases reported, no established treatment is available. And metastatic spread to the small intestine from a tumor originating outside the peritoneal cavity is rare. However, there is a higher tendency for metastasis to the peritoneal cavity, including the small bowel, in the case of lobular carcinoma of the breast.

**CASE PRESENTATION:**

A 72-year-old man who initially presented with complaints of abdominal distention. Computed tomography revealed small bowel stenosis. Post-endoscopic stenosis dilatation, an emergency small bowel resection was performed for small bowel perforation. Postoperative histopathology revealed small bowel metastasis due to mammary gland lobular carcinoma with human epidermal growth factor receptor 2 (3+), estrogen receptor-negative, and progesterone receptor-negative status; the patient was then referred to our hospital. Imaging examinations revealed multiple lymph node metastases in the cervical region, right supraclavicular area, mediastinum, hilar region, and splenic portal. However, no obvious breast lesions or axillary lymph node metastases were identified, leading to a diagnosis of metastatic occult breast cancer. Complete response was achieved with trastuzumab plus pertuzumab plus docetaxel therapy; 30 months after chemotherapy initiation, multiple brain metastases were detected. Thus, 30 Gy whole-brain radiotherapy was performed followed by second-line treatment with trastuzumab emtansine. The patient died 4 years and 8 months after the disease onset, due to the progression of the disease with the new brain metastases.

**CONCLUSIONS:**

For male occult breast cancer, it is important to understand the potential metastatic patterns and genetic factors, as well as to utilize comprehensive diagnostic methods for early diagnosis and disease management.

## Abbreviations

AKT1
: AKT serine/threonine kinase 1
ALND
: axillary lymph node dissection
BM
: brain metastases
*BRCA*
: breast cancer susceptibility genes
CA 15-3
: carbohydrate antigen 15-3
CDH1
: cadherin 1
CEA
: carcinoembryonic antigen
CK
: cytokeratin
DOC
: docetaxel
ER
: estrogen receptor
ERBB2
: erb-b2 receptor tyrosine kinase 2
ESR1
: estrogen receptor 1
FOXA1
: forkhead box protein A1
GATA3
: GATA-binding protein-3
GCDFP-15
: gross cystic disease fluid protein-15
GM
: gastrointestinal metastases
HBOC
: hereditary breast and ovarian cancer syndrome
HER2
: human epidermal growth factor receptor 2
IDC
: invasive ductal carcinoma
IGFR1
: insulin-like growth factor receptor 1
ILC
: invasive lobular carcinoma
LFS
: Li-Fraumeni syndrome
OBC
: occult breast cancers
Per
: pertuzumab
PR
: progesterone receptor
SUVmax
: maximum standardized uptake value
T-DM1
: trastuzumab emtansine
TP53
: tumor protein p53
Tr
: trastuzumab
WBRT
: whole-brain radiation therapy

## INTRODUCTION

OBC, first reported by Halsted in 1907, is a rare entity characterized by axillary lymph nodes or distant organ metastases without identifiable primary breast lesions.^1)^ Most OBC involve axillary lymph node metastases, with distant metastases such as to the skin or brain rarely reported.^2,3)^ However, in some cases of OBC, metastases can appear in unexpected organs, including the small bowel. This occurrence is more commonly seen in metastatic breast cancer, particularly ILC of the breast.^4,5)^ Despite the developments in breast imaging, the incidence of OBC has not decreased; representing <1% of all breast cancers, and male patients account for only about 1% of OBC cases.^6)^ In this report, we demonstrate a case of male OBC with small bowel metastasis presenting obstruction.

## CASE PRESENTATION

A 72-year-old man presented to his family doctor with complaints of abdominal distention lasting for about 2 months, accompanied by nausea, loss of appetite, and weight loss. CT revealed small bowel stenosis with proximal distension. Emergency small bowel resection was performed for a small perforation that developed after endoscopic stenosis dilatation. There was no family history of breast cancer or of other tumors; neither was there any sign of exposure to epidemiologic risk factors. Additionally, there was no notable past history suggesting a hereditary disease.

**Fig. 1:**
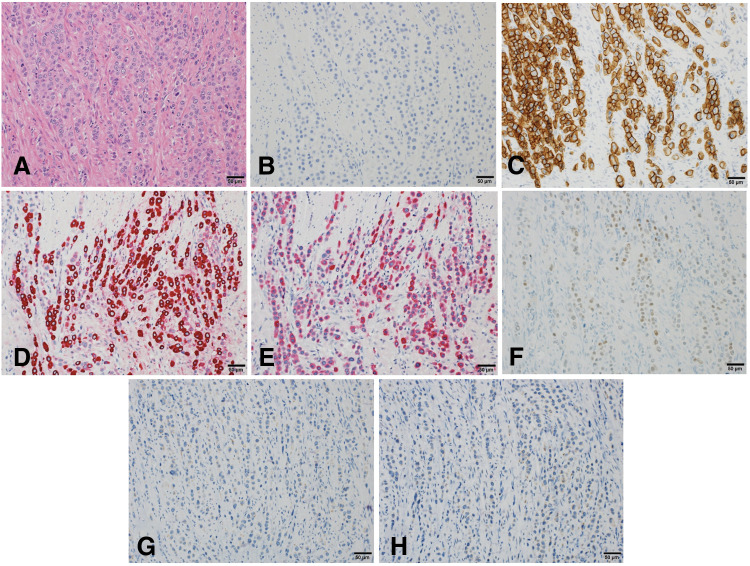
Histopathological examination-immunohistochemistry (Scale bar = 50 μm). (**A**) Hematoxylin-Eosin: Pale eosinophilic cytoplasmic tumor cells with decreased cellular adhesion. (**B**) E-cadherin: negative. (**C**) Human epidermal growth factor receptor type 2: positive (3+). (**D**) Mammaglobin: positive. (**E**) Gross cystic disease fluid protein-15: positive. (**F**) GATA-binding protein-3: positive. (**G**) Estrogen receptor: negative, (**H**) Progesterone receptor: negative.

Postoperative pathological examination (**Fig. 1A**–**1H**) revealed invasion of cancer cells from the serosal surface of the small bowel. The tumor cells had pale eosinophilic cytoplasm and decreased cellular adhesion. Immunohistochemically, tumor cells were negative for ER, PR, and E-cadherin, but positive for HER2 (3+), mammaglobin, GCDFP-15, and GATA3. The patient was identified as ILC of the breast and was subsequently referred to our hospital for further evaluation and treatment.

**Fig. 2:**
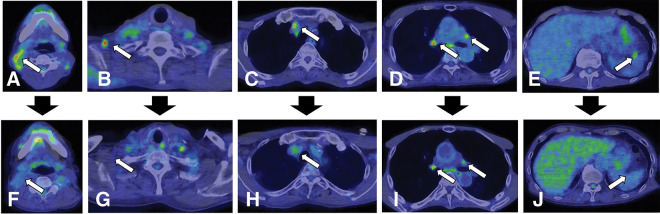
Positron emission tomography scan (**A**–**E**: before chemotherapy; **F**–**J**: after chemotherapy). (**A**–**D**) Abnormal accumulation as indicated by maximum standardized uptake value (SUVmax) in cervical lymph node, right supraclavicular lymph nodes, mediastinum, and hilar region (SUVmax = 2.8–7.1) (arrows). (**E**) Abnormal accumulation as indicated by SUVmax = 3.9 at the splenic portal (arrow). (**F**–**J**) Loss of accumulation or reactive accumulation (arrows).

Blood serum levels of CEA and CA 15-3 were found to be elevated. Despite the unavailability of routine mammography for this extremely slender patient with smaller male breasts containing less glandular tissue, breast ultrasound, contrast-enhanced CT, and PET scan revealed no obvious neoplastic lesions or enlarged lymph nodes in the breast or axilla (**Supplementary Fig. 1A**–**1H**). PET scan (**Fig. 2A**–**2E**) showed multiple accumulation areas in the cervical lymph node, right supraclavicular lymph nodes, mediastinum, and pulmonary hilum (SUVmax = 2.8–7.1); abnormal accumulation was also observed in the splenic hilum (SUVmax = 3.9). Endoscopic examination revealed no esophago-gastrointestinal neoplastic lesion. Thus, we diagnosed the current case as OBC with multiple organ metastases.

**Fig. 3:**
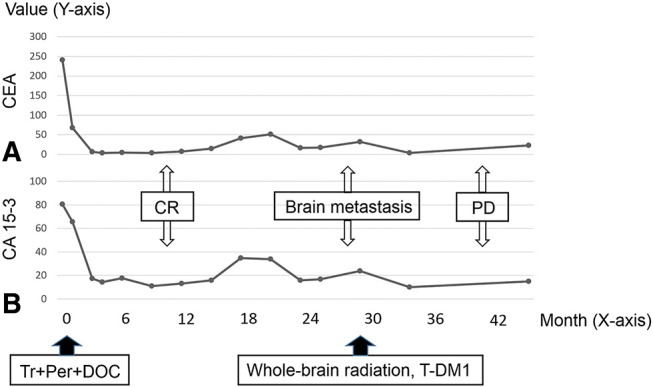
Treatment progress. (**A**) Carcinoembryonic antigen value transition. (**B**) Carbohydrate antigen 15-3 value transition. CA 15-3, carbohydrate antigen 15-3; CEA, carcinoembryonic antigen; CR, complete response; DOC, docetaxel; PD, progressive disease; Per, pertuzumab; T-DM1, trastuzumab emtansine; Tr, trastuzumab

**Fig. 4:**
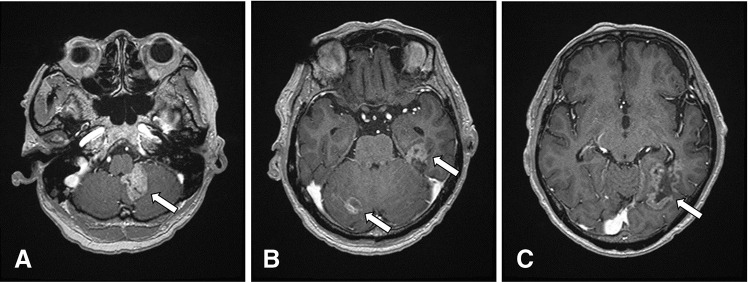
Head magnetic resonance imaging after neurological symptoms. Multiple enhancing lesions within the brain (arrows). (**A**) Cerebellum near left foramen of Luschka. (**B**) Hippocampus, right cerebellum. (**C**) Temporal-occipital lobe around the left lateral ventricle.

**Fig. 5:**
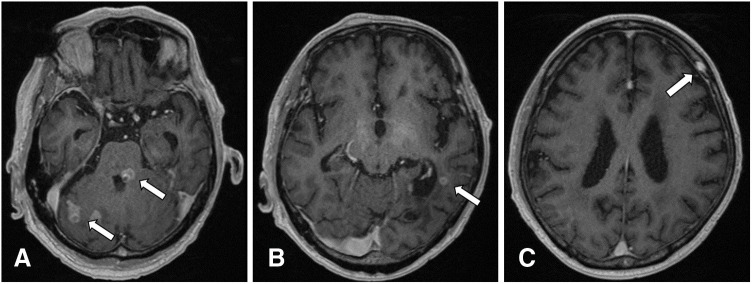
Head magnetic resonance imaging after secondary treatment. Multiple new lesions within the brain (arrows). (**A**) Right and left-brain ventricles. (**B**) Near left lateral ventricle. (**C**) Left frontal periosteum.

The patient’s progression is shown in **Fig. 3**. The patient was administered trastuzumab (Tr: Initial dose of 8 mg/kg followed by 6 mg/kg) plus pertuzumab (Per: Initial dose of 840 mg followed by 420 mg) plus docetaxel (DOC: 60 mg/m^2^) therapy in a 3-week cycle. PET scan obtained after 12 cycles showed that all areas of abnormal accumulation had disappeared or improved (**Fig. 2F**–**2J**), both serum levels of CEA and CA15-3 were normalized, and the patient achieved clinical complete response. At 18 months post-onset of chemotherapy, the serum levels of CEA and CA 15-3 increased once more (**Fig. 3**); however, no abnormal findings during the follow-up imaging were revealed. At 30 months after chemotherapy initiation, the patient experienced vertigo and lightheadedness, which worsened over time with the onset of neurological symptoms such as visual field disturbance and difficulty in walking. The head MRI (**Fig. 4A**–**4C**) showed multiple enhancing lesions in the left cerebellum, hippocampus, right cerebellum, and temporal-occipital lobe areas around the left temporal ventricle, near the left foramen of Luschka, which were diagnosed as multiple BM. For the treatment of multiple BM, the patient and his family opted not to pursue stereotactic radiosurgery; therefore, WBRT was chosen. After receiving second-line treatment with 30 Gy WBRT and 14 cycles of trastuzumab emtansine (T-DM1: 3.6 mg/kg, every 3 weeks), his neurological symptoms, activities of daily living, and the tumor markers showed improvement. However, head MRI (**Fig. 5A**–**5C**) revealed that although the originally noted metastases tended to shrink, new lesions had appeared in the left and right portions of the cerebellum, near the left lateral ventricle, and in the left frontal periosteum. The patient had a progressive disease, and palliative care with symptomatic treatment was provided based on the wishes of the patient and his family. He died 4 years and 4 months after treatment onset.

## DISCUSSION

Male OBC is extremely rare worldwide, making diagnosis challenging. Therefore, identifying metastatic sites using endoscopic and imaging methods, evaluating the primary site through histological examination with immunohistochemical analyses, and providing treatment based on the organ of origin are essential. Immunohistochemically, CK7 and CK20 are often used in the estimation of the primary organ, and addition of tissue-specific markers provide a higher estimation rate of the primary tumor. Generally, positive results for ER, PR, and HER2 staining support primary breast cancer diagnosis; however, this specificity is not sufficient. In addition to these markers, mammaglobin, GCDFP-15, GATA3, and lactalbumin are also useful in supporting the diagnosis of primary breast cancer. This case was diagnosed as OBC based on immunostaining results. Additionally, negative E-cadherin expression and HER2-positive findings suggested ILC.

The drug treatment strategies for ILC are generally recommended to follow the same protocols as those for typical invasive breast cancer.^7)^ While ILC is considered to have a relatively favorable prognosis compared with IDC, it is also reported to have a higher incidence of late recurrences.^8)^ Therefore, careful long-term follow-up is necessary for ILC patients. In terms of imaging diagnostics, MRI has been reported to have the highest sensitivity, ranging from 93% to 100%.^9)^ Additionally, several cohorts suggest that PET/CT modalities using 18F-fluciclovine or 18F-fluoroestradiol may be superior in detecting ILC metastases.^10,11)^

The mechanisms of metastasis in ILC remain unclear, though recent studies have identified potential correlations with its metastatic behavior. Immunophenotypic analyses have shown diverse interactions between hormone receptors and their cofactors as cancer progresses, particularly highlighting frequent downregulation of PR expression and variability in androgen receptor, GATA3, and FOXA1 across different metastatic sites within the same patient.^12)^ Additionally, several cohort studies have indicated an increased frequency of gene mutations, CDH1, AKT1, ESR1, IGFR1, and ERBB2, in metastatic ILC.^13,14)^ ILC metastasizes not only to common sites but also frequently to the peritoneum, ovaries, and the gastrointestinal tract.^5)^ Recognizing these patterns is crucial for the early diagnosis and effective disease management of ILC.

This case was diagnosed as male OBC based on histopathological findings, but the possibility of HBOC cannot be entirely ruled out. Although there is no notable family history or past history that would strongly suggest HBOC in this case, the association between HBOC and male breast cancer has been recognized, and it cannot be dismissed that this case may have had pathogenic *BRCA* 1/2 mutations.^15)^

In addition to HBOC, LFS is another hereditary condition associated with breast cancer. LFS is caused by germline TP53 mutations and is known to increase the risk of developing breast cancer.^16)^ Furthermore, it has been suggested that breast cancers arising in the context of pathogenic TP53 mutations are more likely to exhibit HER2 amplification.^17)^ Considering the rarity of HER2-positive subtype male breast cancer patients, it cannot be ruled out that this case may have harbored a pathogenic TP53 mutation.

Male breast cancer is generally known to have a very high ER and PR positivity rate (more than 80%) and a low HER2 positivity rate.^18)^ However, this case was a very rare immunophenotype with ER and PR-negative and HER2-positive. To our knowledge, this is the fifth case of a HER2-positive male OBC (**Table 1**).^19–22)^ Three of the 4 previously reported cases involved only axillary lymph node metastasis, and only 1 case involved distant skin metastasis. All M0 cases underwent at least mastectomy, ALND, or WBRT, resulting in disease control. However, unlike most of the previously reported cases, our case was not accompanied by axillary lymph node metastasis but involved the small bowel at diagnosis and was a stage IV disease.

**Table 1:** Reported HER2-positive male occult primary breast cancers

Study/reference year	Age	TNM classification	Subtype	Local treatment	Adjuvant therapy	Recurrence	OS (months)	Prognosis
Ogata et al., 2025 (present case)	72	T0N3M1 (Stage Ⅳ)	ER(−), PR(−), HER2 (3+)	Small bowel resection without ALND or mastectomy	Molecular targeted therapy, Chemotherapy, Radiotherapy	Brain	52	Death
Xu et al., 2017^19)^	29	T0N3M0 (Stage IIIC)	ER(+), PR(+), HER2 (3+)	ALND and whole breast radiotherapy without mastectomy	Chemotherapy	None	19	Survival
Kuninaka et al., 2017^20)^	67	T0N3M1 (Stage Ⅳ)	ER(+), PR(+), HER2 (3+)	Skin biopsy without ALND or mastectomy	Molecular targeted therapy	None	33	Survival
He et al., 2015^21)^	40	T0N3M0 (Stage IIIC)	ER(−), PR(+), HER2 (2+), FISH(+)	Mastectomy with ALND	Endocrinotherapy, Chemotherapy	Lung and lymph nodes (Axillary and supraclavicular)	27	Survival
Gu et al., 2009^22)^	72	T0N2M0 (Stage IIIA)	ER(−), PR(−), HER2 (3+)	Mastectomy with ALND	None	None	24	Survival

ALND, axillary lymph node dissection; ER, estrogen receptor; FISH, fluorescent *in situ* hybridization; HER2, human epidermal growth factor receptor 2; OS, overall survival; PR, progesterone receptor

HER2-positive breast cancer is known to have a higher incidence of BM compared with other subtypes.^23)^ For cases of BM, the efficacy of drugs such as tucatinib and trastuzumab deruxtecan has been suggested, and recent advancements in systemic therapy have significantly improved clinical outcomes.^7,24)^

GM from breast cancer occur in approximately 10% of all metastatic cases, with few reports on male breast cancer.^25)^ The reported case we identified involved hormone receptor-positive and HER2-positive IDC, whereas our case presented as hormone receptor-negative and HER2-positive ILC.^26)^ It is known that GM are more commonly derived from lobular carcinoma than from ductal carcinoma.^5)^ Furthermore, a significant difference between the reported case and the present case is that the present case involved male OBC.

Due to its rarity, male OBC is often advanced by the time symptoms appear. As in the present case, where metastases are observed at nonspecific sites at the time of diagnosis, distinguishing them as breast cancer becomes more challenging. Given this context, we cannot rule out the possibility that some cases of male OBC may be treated as cancers of unknown primary origin. To effectively screen for such cases, it is crucial for clinicians to understand the characteristics of male OBC, develop research designs and guidelines specific to this condition, and standardize the diagnostic and treatment processes.

## CONCLUSIONS

We report a case of male OBC. For male OBC, it is important to understand the potential metastatic patterns and genetic factors, as well as to utilize comprehensive diagnostic methods for early diagnosis and disease management.

## SUPPLEMENTARY MATERIALS

Supplementary Fig. 1 Imaging findings at diagnosis. No abnormal findings were observed in the breast or axillary lymph node area on ultrasound examination (**a–d**), contrast-enhanced computed tomography scan (**e**, **f**), or positron emission tomography scan (**g**, **h**).

## ACKNOWLEDGMENTS

The authors thank Editage (www.editage.jp) for English language editing.

## DECLARATIONS

### Funding

Not applicable.

### Authors’ contributions

SO: design concept, investigation, acquisition of data, drafting, and revision of the article.

UT: design concept, revision and editing, and supervision.

KO: acquisition of data and revision and editing.

YM: diagnosis of pathological histology and revision of the manuscript.

NO: acquisition of data and revision and editing.

YT: revision and editing.

SS: revision and editing.

RS: acquisition of data and revision and editing.

FF: revision and editing and supervision.

All authors have read and approved the final manuscript.

### Availability of data and materials

The data that support the findings of this study are available from the corresponding author upon reasonable request.

### Ethics approval and consent to participate

This work does not require ethical considerations or approval. Informed consent to participate in this study was obtained from the patient.

### Consent for publication

Written informed consent for publication of the case details and images was obtained from the patient.

### Competing interests

The authors declare that they have no competing interests.
