# Supplementary figures and images for: Male Occult Primary Breast Cancer Diagnosed with Small Bowel Metastases: A Case Report
Source: Surg Case Rep. 2025 May 16;11(1):24-0089. doi: 10.70352/scrj.cr.24-0089 (PMC12105989; doi:10.70352/scrj.cr.24-0089)

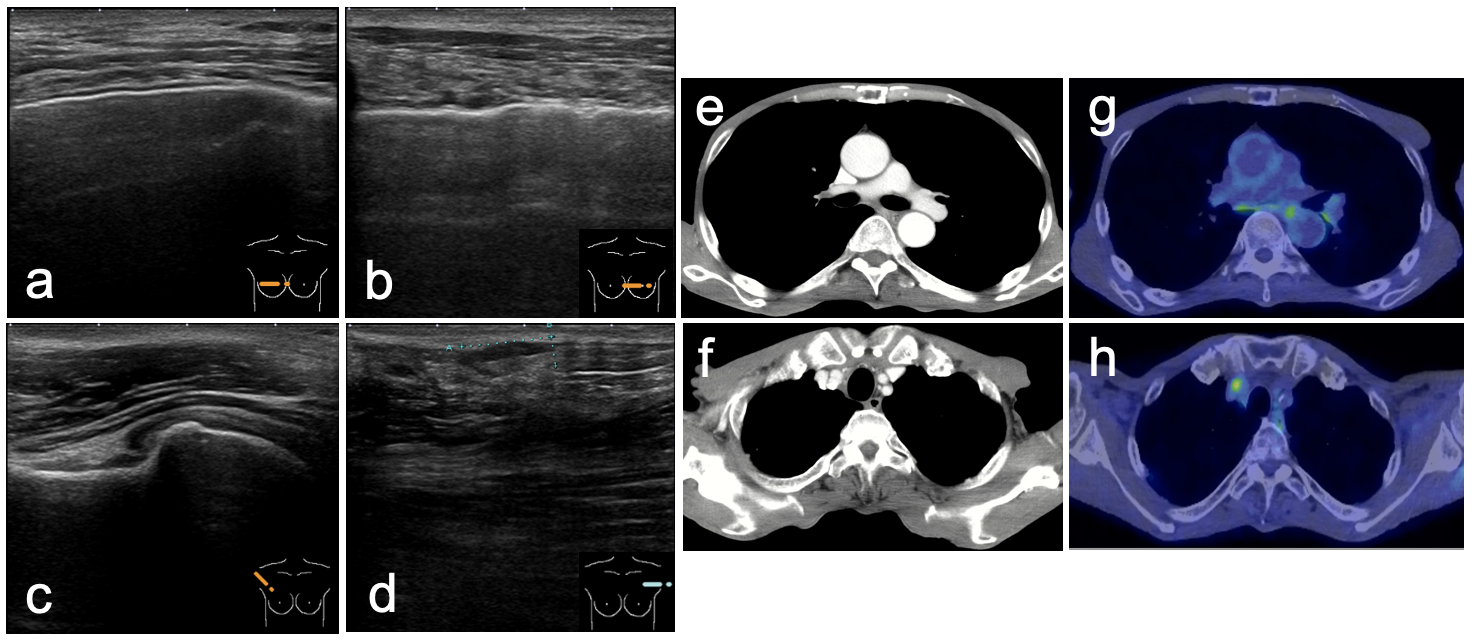

Supplement: Supplementary Fig. 1 [file scr-11-01-24-0089-s001.tiff]
